# Transcatheter Aortic Valve Replacement with Self-Expandable ACURATE neo as Compared to Balloon-Expandable SAPIEN 3 in Patients with Severe Aortic Stenosis: Meta-Analysis of Randomized and Propensity-Matched Studies

**DOI:** 10.3390/jcm9020397

**Published:** 2020-02-01

**Authors:** Mirosław Gozdek, Kamil Zieliński, Michał Pasierski, Matteo Matteucci, Dario Fina, Federica Jiritano, Paolo Meani, Giuseppe Maria Raffa, Pietro Giorgio Malvindi, Michele Pilato, Domenico Paparella, Artur Słomka, Jacek Kubica, Dariusz Jagielak, Roberto Lorusso, Piotr Suwalski, Mariusz Kowalewski

**Affiliations:** 1 Department of Cardiology and Internal Medicine, Nicolaus Copernicus University, Collegium Medicum, 85067 Bydgoszcz, Poland; gozdekm@wp.pl (M.G.); kubicajw@gmail.com (J.K.); 2 Thoracic Research Centre, Nicolaus Copernicus University, Collegium Medicum in Bydgoszcz, Innovative Medical Forum, 85067 Bydgoszcz, Poland; kamilziel@gmail.com (K.Z.); michalpasierski@gmail.com (M.P.);; 3 Department of Cardiology, Warsaw Medical University, 02091 Warsaw, Poland; 4 Clinical Department of Cardiac Surgery, Central Clinical Hospital of the Ministry of Interior and Administration, Centre of Postgraduate Medical Education, 02607 Warsa, Poland; suwalski.piotr@gmail.com (P.S.); 5 Department of Cardio-Thoracic Surgery, Heart and Vascular Centre, Maastricht University Medical Centre, 6229 HX Maastricht, The Netherlands; teo.matte@libero.it (M.M.); dario.fina88@gmail.com (D.F.); fede.j@hotmail.it (F.J.); paolo.meani@ospedaleniguarda.it (P.M.); roberto.lorussobs@gmail.com (R.L.); 6 Department of Cardiac Surgery, Circolo Hospital, University of Insubria, 21100 Varese, Italy; 7 Department of Cardiology, IRCCS Policlinico San Donato, University of Milan, 20097 Milan, Italy; 8 Department of Cardiac Surgery, University Magna Graecia of Catanzaro, 88100 Catanzaro, Italy; 9 Department of Intensive Care Unit, Maastricht University Medical Centre (MUMC+), 6229 HX Maastricht, The Netherlands; 10 Department for the Treatment and Study of Cardiothoracic Diseases and Cardiothoracic Transplantation, IRCCS-ISMETT (Instituto Mediterraneo per i Trapianti e Terapie ad alta specializzazione), 90127 Palermo, Italy; giuseppe.raffa78@gmail.com (G.M.R.); mpilato@ISMETT.edu (M.P.); 11 Wessex Cardiothoracic Centre, University Hospital Southampton, Southampton SO16 6YD, UK; pg.malvindi@hotmail.com; 12 GVM Care & Research, Department of Cardiovascular Surgery, Santa Maria Hospital, 70124 Bari, Italy; domenico.paparella@uniba.it; 13 Department of Emergency and Organ Transplant, University of Bari Aldo Moro, 70121 Bari, Italy; 14 Chair and Department of Pathophysiology, Nicolaus Copernicus University, Collegium Medicum, 85067 Bydgoszcz, Poland; 15 Department of Cardiac Surgery, Gdańsk Medical University, 80210 Gdańsk, Poland; kardchir@gumed.edu.pl

**Keywords:** meta-analysis, ACURATE neo, SAPIEN 3, transcatheter aortic valve replacement

## Abstract

Frequent occurrence of paravalvular leak (PVL) after transcatheter aortic valve replacement (TAVR) was the main concern with earlier-generation devices. Current meta-analysis compared outcomes of TAVR with next-generation devices: ACURATE neo and SAPIEN 3. In random-effects meta-analysis, the pooled incidence rates of procedural, clinical and functional outcomes according to VARC-2 definitions were assessed. One randomized controlled trial and five observational studies including 2818 patients (ACURATE neo *n* = 1256 vs. SAPIEN 3 *n* = 1562) met inclusion criteria. ACURATE neo was associated with a 3.7-fold increase of moderate-to-severe PVL (RR (risk ratio): 3.70 (2.04–6.70); *P* < 0.0001), which was indirectly related to higher observed 30-day mortality with ACURATE valve (RR: 1.77 (1.03–3.04); *P* = 0.04). Major vascular complications, acute kidney injury, periprocedural myocardial infarction, stroke and serious bleeding events were similar between devices. ACURATE neo demonstrated lower transvalvular pressure gradients both at discharge (*P* < 0.00001) and at 30 days (*P* < 0.00001), along with lower risk of patient–prosthesis mismatch (RR: 0.29 (0.10–0.87); *P* = 0.03) and pacemaker implantation (RR: 0.64 (0.50–0.81); *P* = 0.0002), but no differences were observed regarding composite endpoints early safety and device success. In conclusion, ACURATE neo, as compared with SAPIEN 3, was associated with higher rates of moderate-to-severe PVL, which were indirectly linked with increased observed 30-day all-cause mortality.

## 1. Introduction

Since first its mention by Cribier in 2002 [1], transcatheter aortic valve replacement (TAVR) has been complementary method to surgical aortic valve replacement (SAVR) in inoperable or high-risk patients with severe symptomatic aortic stenosis. Similar [2] or even lower [3] one-year mortality rate of TAVR, as compared to SAVR, was shown in selected groups of patients. Hence, TAVR is now considered to be an alternative treatment option and is recommended not only in inoperable, high or increased risk surgical patients [2,3,4,5] but also in intermediate and lower risk individuals [6,7,8,9,10]. Commercially available earlier-generation transcatheter valves, despite providing good clinical outcomes, were not free from shortcomings; indeed, high rates of conduction abnormalities, permanent pacemaker implantation (PPI) or vascular complications remained important issues to be addressed. More importantly, though, higher incidence of paravalvular leak (PVL), in turn associated with increased late mortality and higher rate of other adverse clinical incidents, as compared to SAVR [11,12,13], often outweigh the benefits of transcatheter approach.

To minimize these shortcomings, technological innovations were developed in next-generation valves including the following: balloon-expandable SAPIEN 3 (Edwards Lifesciences, Irvine, CA, USA) and self-expandable ACURATE neo (Boston Scientific Corporation, Marlborough, MA, USA). Since direct comparisons of these two devices are few and one recent randomized controlled trial (RCT) [14] did not demonstrate non-inferiority of the ACURATE neo device as compared to SAPIEN 3 as opposed to previous observational studies [15,16,17,18,19,20,21] that, however, pointed to comparable or superior results with ACURATE, the debate is ongoing. 

The objective of the present investigation was to evaluate and compare short-term results of TAVR with ACURATE neo and SAPIEN 3 in patients presenting with symptomatic severe native aortic valve stenosis.

## 2. Experimental Section

### 2.1. Data Sources and Search Strategy

The systematic review and meta-analysis were performed in accordance to MOOSE statement and PRISMA guidelines [22,23]. The MOOSE checklist is available as Table A1. We searched PubMed, ClinicalKey, the Web of Science and Google Scholar all until October 2019. Search terms were as follows: “ACURATE neo” (or “ACCURATE neo”), “Symetic ACURATE”, “Boston ACURATE” and/or “SAPIEN 3”, “SAPIEN III” and “transcatheter valve” or “aortic”. The literature was limited to peer-reviewed articles published in English. References of original articles were reviewed manually and cross-checked. 

### 2.2. Selection Criteria and Quality Assessment

Studies were included if having met all of the following criteria: (1) human study; (2) study or study arms comparing directly strategy of transcatheter aortic valve replacement with ACURATE neo and SAPIEN 3; (3) RCT or propensity score matched observational study. Studies were excluded if they fell into the following categories: (1) in-vitro study; (2) single arm; (3) adjustment not PS or methods not reported; (4) outcomes of interest not reported; and (5) sub-studies or overlapping populations. No restrictions regarding number of patients included or characteristic of the population were imposed. Two reviewers (M.G. and K.Z.) selected the studies for the inclusion, extracted studies and patients’ characteristics of interest and relevant outcomes. Two authors (M.G. and K.Z.) independently assessed the trials’ eligibility and risk of bias. Any divergences were resolved by consensus.

Quality of RCTs was appraised by using the components recommended by the Cochrane Collaboration [24]; observational studies were, instead, appraised with ROBINS-I (Risk of Bias in Nonrandomised Studies-of Interventions), a tool used for assessment of the bias (the selection of the study groups; the comparability of the groups; and the ascertainment of either the exposure or outcome of interest) in cohort studies included in a systematic review and/or meta-analysis [25].

### 2.3. Endpoints Selection

Endpoints were established according to the Valve Academic Research Consortium-2 (VARC-2) definitions [26]. Procedural outcomes of interest were predilatation and postdilatation, procedural times and contrast volume. Clinical endpoints assessed included the following: PPI, major vascular complications (MVC), serious bleeding (life-threatening and/or major), acute kidney injury (AKI), stroke, myocardial infarction and 30-day mortality. Functional outcomes were as follows: mean transvalvular gradients, prosthesis-patient mismatch (PPM), and mild and moderate-to-severe paravalvular leak (PVL). Composite endpoints were as per VARC-2: device success (defined as absence of procedural death, correct position of 1 valve in the proper location, mean gradient < 20 mm Hg or peak velocity < 3 m/s, absence of moderate-to-severe PVL and absence of PPM) and early safety (composite of all-cause death, any stroke, life-threatening or disabling bleeding, major vascular complications, coronary artery obstruction requiring intervention, acute kidney injury (stage 2 or higher), rehospitalization for valve-related symptoms or congestive heart failure, valve-related dysfunction requiring repeat procedure, and valve-related dysfunction determined by echocardiography (mean aortic valve gradient ≥ 20 mm Hg and either effective orifice area ≤ 0.9–1.1 cm² (depending on body surface area) or Doppler velocity index < 0.35; or moderate or severe prosthetic PVL).

### 2.4. Statistical Analysis

Data were analyzed according to intention-to-treat principle, wherever applicable. Risks ratios (RR) and 95% confidence intervals (95% CI) served as primary index statistics for dichotomous outcomes. For continuous outcomes, mean difference (MD) and corresponding 95% CI were calculated by using a random effects model. To overcome the low statistical power of Cochran Q test, the statistical inconsistency test *I*^2^ = [(Qdf)/Q]×100%, where Q is the chi-square statistic and df is its degrees of freedom, was used to assess heterogeneity [27]. It examines the percentage of inter-study variation, with values ranging from 0% to 100%. An *I*^2^ value of 25% indicates low heterogeneity, 50% are suggestive of moderate heterogeneity and 70% of high heterogeneity. Because of high degree of heterogeneity anticipated among predominantly nonrandomized trials, an inverse variance (DerSimonian–Laird) random-effects model was applied as a more conservative approach for observational data accounting for between- and within-study variability. Whenever a single study reported median values and interquartile ranges instead of mean and standard deviation (SD), the latter were approximated as described by Wan and colleagues [28]. In case there were “0 events” reported in both arms, calculations were repeated, as a sensitivity analysis, using risk difference (RD) and respective 95% CI. Additionally, we performed a set of meta-regression analyses to address potential relationships between 30-day all-cause mortality and other endpoints and baseline characteristics assessed. For the analyses of clinical endpoints, RCTs and PS-matched studies were analyzed separately. Review Manager 5.3 (The Nordic Cochrane Centre, Copenhagen, Denmark) was used for statistical computations. *P*-values ≤ 0.05 were considered statistically significant and reported as two-sided, without adjustment for multiple comparisons.

## 3. Results

### 3.1. Study Selection and Bias

**Figure 1:**
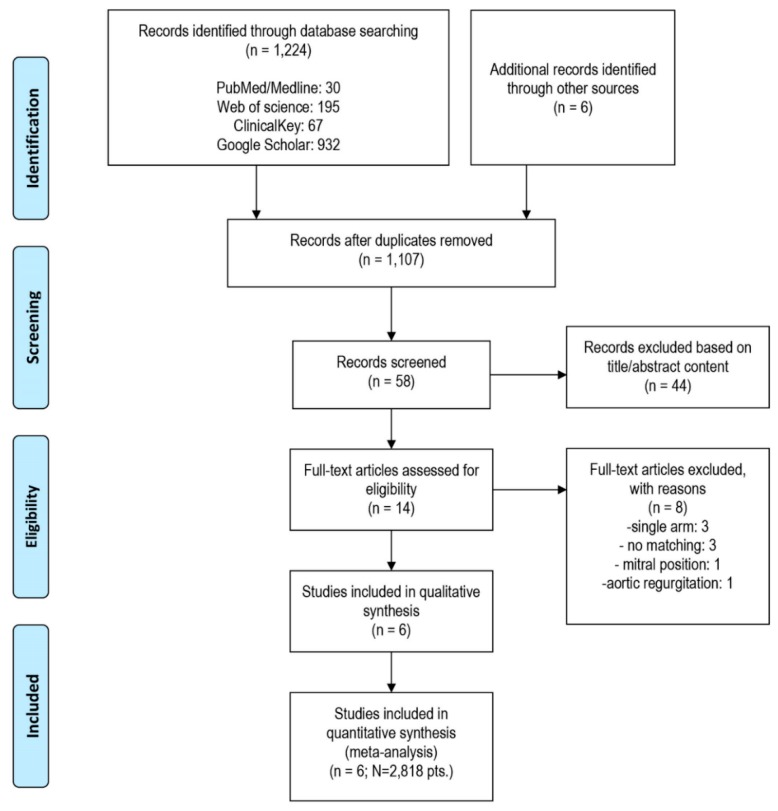
Study selection and inclusion process.

Study selection process and reasons for exclusion of some studies are described in Figure 1.

Systematic search of the online databases allowed collection of 58 potentially eligible records that were retrieved for scrutiny. Of those, 52 were further excluded because they were not pertinent to the design of the meta-analysis or did not meet the explicit inclusion criteria. One RCT [14] and five observational studies [15,16,17,18,19] enrolling the total of 2818 patients were eventually included in the analysis. Potential sources of the studies’ bias were analyzed with the use of components recommended by the Cochrane Collaboration and ROBINS-I tool, and the results are enclosed as Table A2. Overall, the studies reported moderate risk of bias. Most commonly, biases arose from participants selection for the study by designated heart teams and subjective distribution of the participants within the study arms. All but one study [14] lacked a core lab assessment of PVL and central adjudication of clinical events.

Patients were divided into two groups: those treated with ACURATE neo transcatheter valve (*n* = 1256) and SAPIEN 3 transcatheter valve (*n* = 1562). Summary of the valve characteristics is available as Table 1.

Studies’ characteristics, as well as definitions or diagnostic criteria for assessed clinical endpoints, are reported in Table 2. Table A3 lists selection criteria for the procedure and valve, as well as inclusion and exclusion criteria within particular studies. Patients’ baseline characteristics and detailed procedural characteristics are available as Table A4 and Table A5. All studies reported data on 30-day clinical outcomes; three reported Kaplan–Meier estimates of survival at longer-term follow-ups [15,16,18].

### 3.2. Patients Characteristic

**Figure 2:**
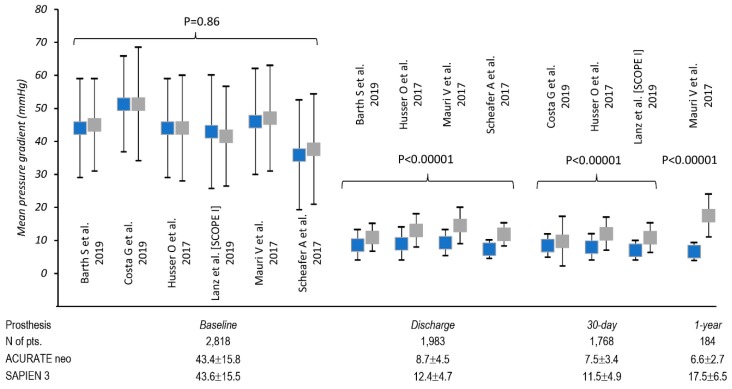
Analysis of mean transaortic gradients before and after transcatheter aortic valve replacement (TAVR).

Groups treated with ACURATE neo and SAPIEN 3 did not differ regarding patients’ age (*P* = 0.363), body mass index (*P* = 0.708), NYHA III/IV status (*P* = 0.115) or left ventricle ejection fraction (*P* = 0.178). No difference was found in the baseline logistic EuroSCORE as well (*P* = 0.749). SAPIEN 3 group included significantly fewer female individuals, 59.7% vs. 64.1%, respectively (*P* = 0.037). Aortic valve baseline echo-parameters, i.e., mean trans-aortic gradient were comparable: 43.4 ± 15.8 vs. 43.6 ± 15.5 mmHg (*P* = 0.861) in ACURATE neo and SAPIEN 3, respectively (Figure 2), although the aortic annulus plane area were on average 4 mm^2^ smaller in the ACURATE neo recipients 439.7 ± 62.4 vs. 446.7 ± 76.3; *P* = 0.037 as compared to SAPIEN 3. Transfemoral access was mostly widely employed during TAVR procedure; in five studies, it was used exclusively [14,16,17,18,19]. Barth et al. [15] included both transfemoral and transapical access in 75.7% vs. 24.3% and 74.5% vs. 25.5% for ACURATE neo and SAPIEN 3, respectively. For the transapical approach, ACUARATE TA device was used.

### 3.3. Procedural Outcomes

**Figure A1:**
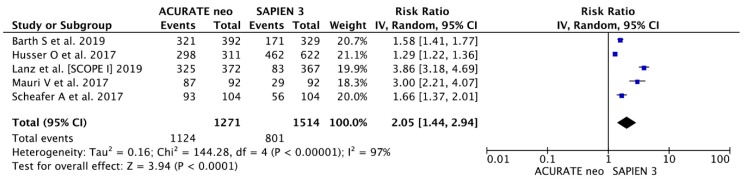
Procedural outcomes. Individual and summary risk ratios with corresponding 95% confidence intervals for the comparison of ACURATE neo vs. SAPIEN 3 in the analysis of predilatation.

**Figure A2:**
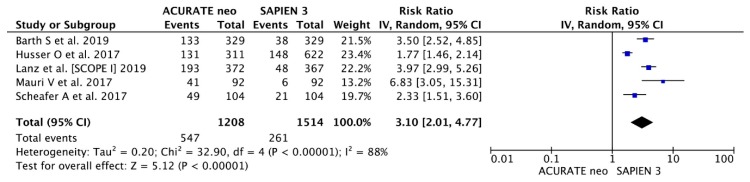
Procedural outcomes. Individual and summary risk ratios with corresponding 95% confidence intervals for the comparison of ACURATE neo vs. SAPIEN 3 in the analysis of postdilatation.

**Figure A3:**
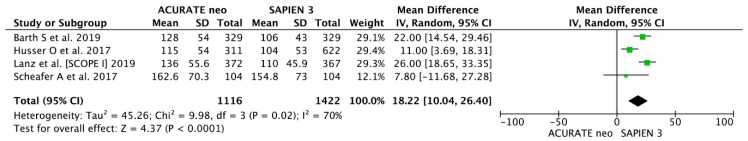
Procedural outcomes. Detailed analysis of individual weighted mean differences (MDs) with corresponding 95% CIs on contrast volume used for the comparison of ACURATE neo vs. SAPIEN 3.

**Figure A4:**
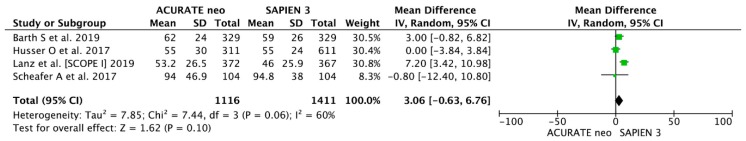
Procedural outcomes. Detailed analysis of individual weighted mean differences (MDs) with corresponding 95% CIs on procedure duration for the comparison of ACURATE neo vs. SAPIEN 3.

Five studies [14,15,17,18,19] and 2722 patients contributed to the analysis of procedural outcomes between two devices. Both predilatation and postdilatation were more common with ACURATE neo valve; predilatation was necessary in 1124/1271 (88.4%) of cases as compared to 801/1514 (52.9%); RR 2.05, 95% CI, (1.44, 2.94) *P* < 0.0001; *I*^2^ = 97%); postdilatation: RR 3.10, 95% CI, (2.01, 4.77) *P* < 0.00001; *I*^2^ = 88%) with respective rates of 45.3% vs. 17.2% for ACURATE neo and SAPIEN 3, respectively. Figure A1 and A2. The procedures performed with ACURATE neo required significantly greater amount of contrast: 130.3 ± 56.1 mL vs. 109.7 ± 50.3 mL (MD 18.22 95% CI, (10.04, 26.40) mL; *P* < 0.0001). Figure A3). Four studies [14,15,17,19] including 1116 ACURATE neo and 1411 SAPIEN 3 cases provided data on procedure duration, which on average 3 minutes longer in the former: 60.1 ± 28.6 min. vs. 56.5.9 ± 26.0 min. (MD 3.06, 95% CI, (−0.66, 6.76) min) without reaching statistical significance (Figure A4). Use of >1 valve was necessary in 35 cases (26 ACURATE neo vs. nine SAPIEN 3; RR 3.24, 95% CI, (1.47, 7.13) *P* = 0.004; *I*^2^ = 0%). Incidence of cardiac tamponade was reported in three studies [14,15,18] with respective event rates of 1.0% vs. 0.7% for ACURATE neo and SAPIEN 3 valves: RR 1.17, 95% CI, (0.52, 2.63) *P* = 0.70; *I*^2^ = 0%. Early procedural complications included the following: coronary obstruction in three ACURATE neo patients and total of eight annular ruptures, 20 conversions to surgery and 20 valve malpositionings without differences between two devices. 

### 3.4. Clinical Outcomes

**Figure 3:**
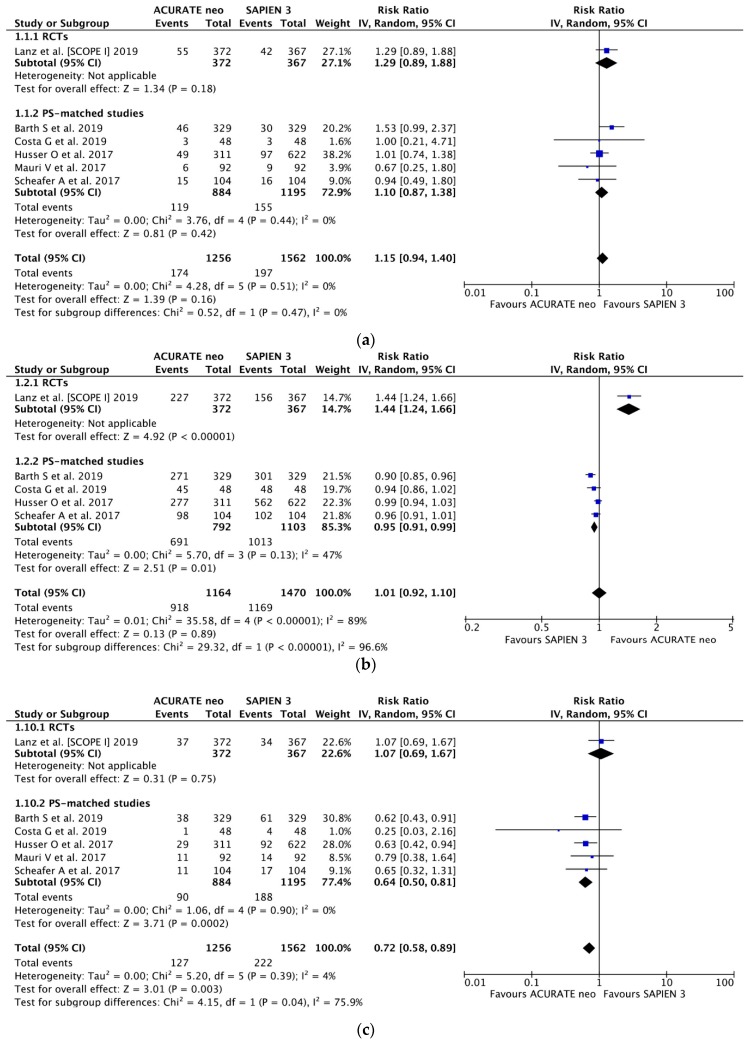
Individual and summary risk ratios with corresponding 95% confidence intervals for the comparison of ACURATE neo vs. SAPIEN 3 in the analysis of clinical outcomes: (**a**) early safety, (**b**) device success and (**c**) permanent pacemaker implantation.

Six studies [14,15,16,17,18,19] enrolling 2818 patients contributed data for the analysis of early safety as defined by VARC-2; with the corresponding rates of 13.9% (174/1256) and 12.6% (197/1562) for ACURATE neo and SAPIEN 3 valves, respectively, there were no statistical differences between two devices (RR 1.15, 95% CI, (0.94, 1.40) *P* = 0.16; *I*^2^ = 0%) and pooled estimates of RCT and PS-matched studies in subgroup analysis (*P*_interaction_ = 0.47) (Figure 3a). In the pooled analysis of device success (five studies included (2634 patients.)), there were no differences between two types of valve in the pooled analysis: RR 1.01, 95% CI, (0.92, 1.10) *P* = 0.89; *I*^2^ = 89%). Analyzed separately, there were strong between-subgroup differences between RCT and pooled estimate from PS-matched studies: RR 1.44, 95% CI, (1.24, 1.66); *P* < 0.00001; *I*^2^ = NA and RR 0.95, 95% CI, (0.91, 0.99); *P* = 0.01; *I*^2^ = 47% with *P*_interaction_ < 0.00001 (Figure 3b).

**Figure A5:**
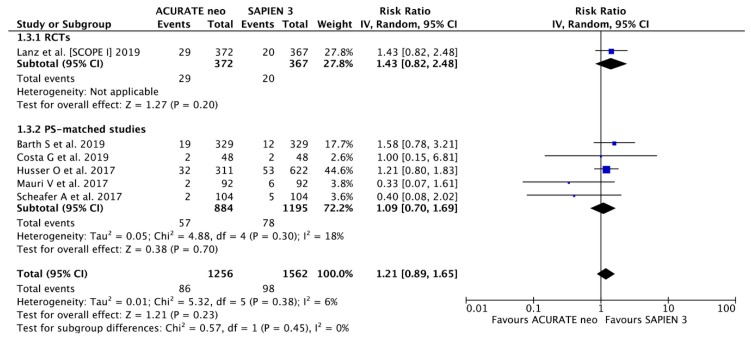
Clinical outcomes. Individual and summary risk ratios with corresponding 95% confidence intervals for the comparison of ACURATE neo and SAPIEN 3 in the analysis of major vascular complications.

**Figure A6:**
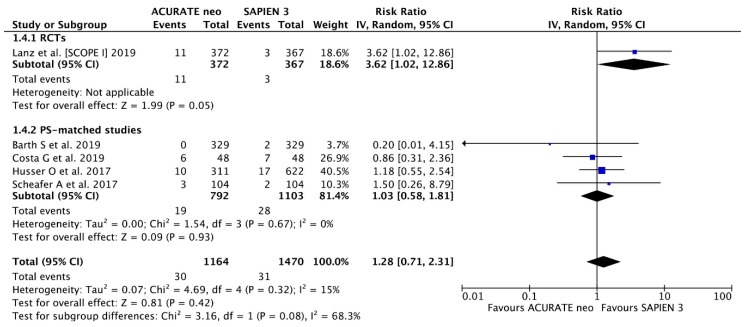
Clinical outcomes. Individual and summary risk ratios with corresponding 95% confidence intervals for the comparison of ACURATE neo and SAPIEN 3 in the analysis of acute kidney injury.

**Figure A7:**
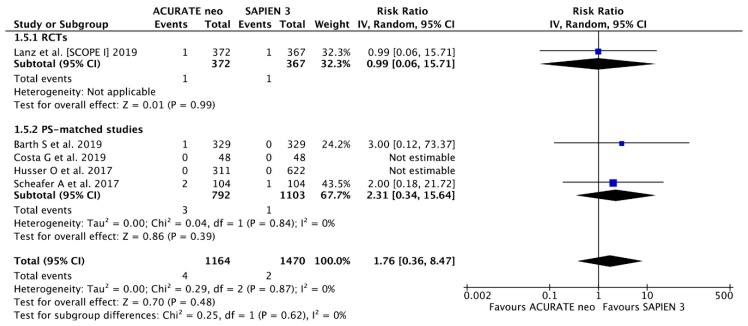
Clinical outcomes. Individual and summary risk ratios with corresponding 95% confidence intervals for the comparison of ACURATE neo and SAPIEN 3 in the analysis of periprocedural myocardial infarction.

**Figure A8:**
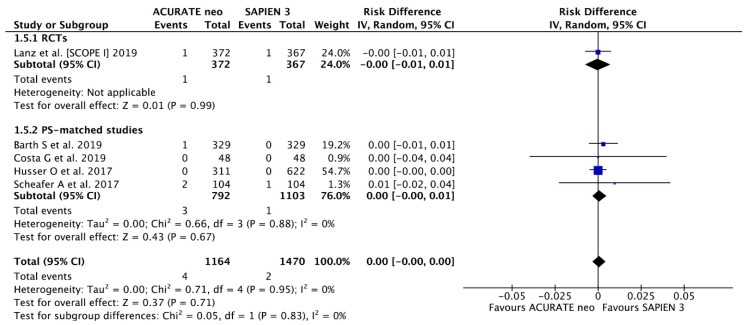
Clinical outcomes. Individual and summary risk ratios with corresponding 95% confidence intervals for the comparison of ACURATE neo and SAPIEN 3 in the analysis of periprocedural myocardial infarction taking into account “0 events”.

**Figure A9:**
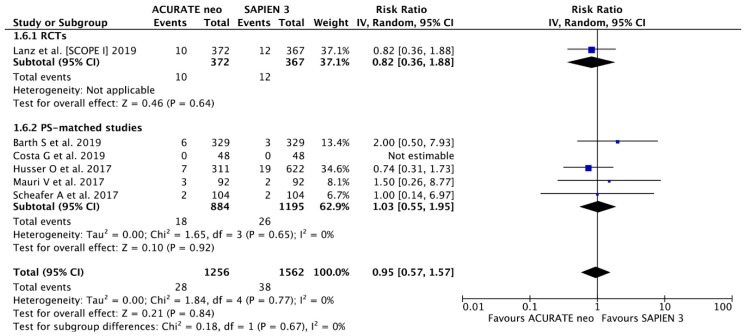
Clinical outcomes. Individual and summary risk ratios with corresponding 95% confidence intervals for the comparison of ACURATE neo and SAPIEN 3 in the analysis of stroke.

**Figure A10:**
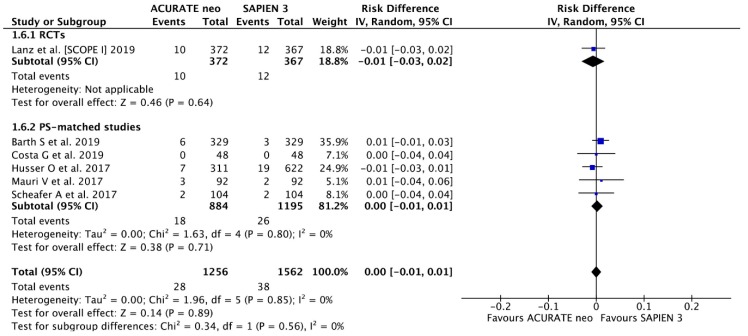
Clinical outcomes. Individual and summary risk ratios with corresponding 95% confidence intervals for the comparison of ACURATE neo and SAPIEN 3 in the analysis of stroke taking into account “0 events”.

**Figure A11:**
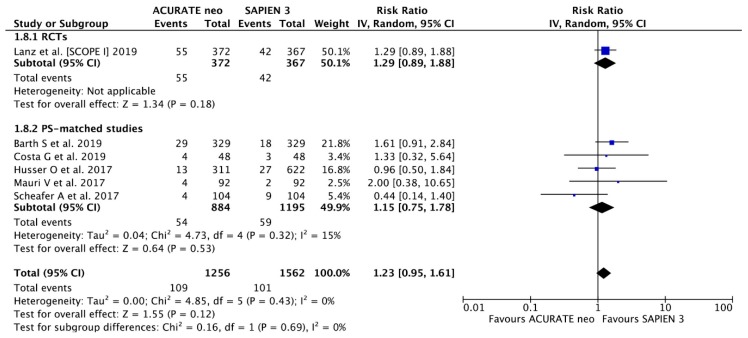
Clinical outcomes. Individual and summary risk ratios with corresponding 95% confidence intervals for the comparison of ACURATE neo and SAPIEN 3 in the analysis serious bleeding events.

There were no differences between ACURATE neo and SAPIEN 3 valves in terms of risk of major vascular complications (RR 1.21, 95% CI, (0.89, 1,65); *P* = 0.23; *I*^2^ = 6%; Figure A5), acute kidney injury (RR 1.28, 95% CI, (0.71, 2,31); *P* = 0.42; *I*^2^ = 15%; Figure A6), periprocedural myocardial infarction (RR 1.76, 95% CI, (0.36, 8.47); *P* = 0.428 *I*^2^ = 0%; Figure A7 and Figure A8), stroke (RR 0.95, 95% CI, (0.57, 1.57); *P* = 0.84 *I*^2^ = 0%; Figure A9 and Figure A10), and serious bleeding events (RR 1.23, 95% CI, (0.95, 1.61); *P* = 0.12; *I*^2^ = 0%; Figure A11).

Based on the data from six studies (2818 pts.), PPI was required nearly 30% less often after ACURATE neo implantation as compared to SAPIEN 3 (RR 0.72, 95% CI, (0.58, 0.89); *P* = 0.003; *I*^2^ = 75.9%) with corresponding frequency of 10.1% vs. 14.2%, respectively (Figure 3c). Importantly, the estimates derived from SCOPE I differed from the pooled estimates (*P*_interaction_ = 0.04) with higher rates of PPI observed in SAPIEN 3 arm in PS-matched studies (9.3% vs. 15.8%) Table A6 lists the VARC-2 derived quality criteria for PPI appraisal 

### 3.5. Functional Outcomes

**Figure 4:**
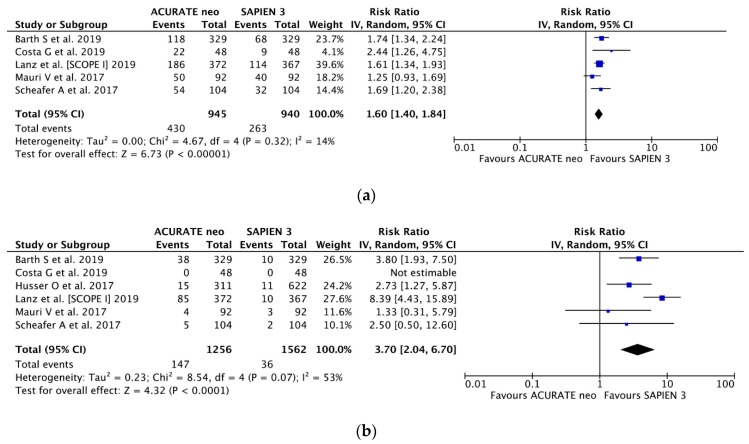
Individual and summary risk ratios with corresponding 95% confidence intervals for the comparison of ACURATE neo vs. SAPIEN 3 in the analysis of functional outcomes: (**a**) mild and (**b**) moderate-to-severe paravalvular leak.

With five studies [14,15,16,18,19] and 1885 patients included, mild PVL occurred less frequently in SAPIEN 3 recipients, 28.0% (263 of 940), compared to ACURATE neo group, 45.5% (430 of 945); (RR 1.60, 95% CI, (1.40, 1.84) *P* < 0.00001; *I*^2^ = 14%) (Figure 4a). Moderate-to-severe PVL was uncommon in the entire series (6.5%); however, there was a significant 3.7-fold increase in moderate-to-severe PVL risk with ACURATE neo implantation: (RR 3.70, 95% CI, (2.04, 6.70) *P* < 0.0001; *I*^2^ = 53%) (Figure 4b) and corresponding incidence of 11.7% (147/1,256) and 2.3% (36/1,562) in ACURATE neo and SAPIEN 3 valves.

Data regarding postprocedural transaortic gradient came from all six studies with 2818 patients. Mauri et al. [18] reported on 1-year transaortic gradients as well. (Figure 2). Mean postprocedural transaortic gradients were higher in SAPIEN 3 patients both at discharge and at 30 days post-op: 12.4 ± 4.7 vs. 8.7 ± 4.5 mmHg (*P* < 0.00001) and 11.5 ± 4.9 vs. 7.5 ± 3.4 mmHg (*P* < 0.00001) respectively.

### 3.6. All-Cause Mortality

**Figure 5:**
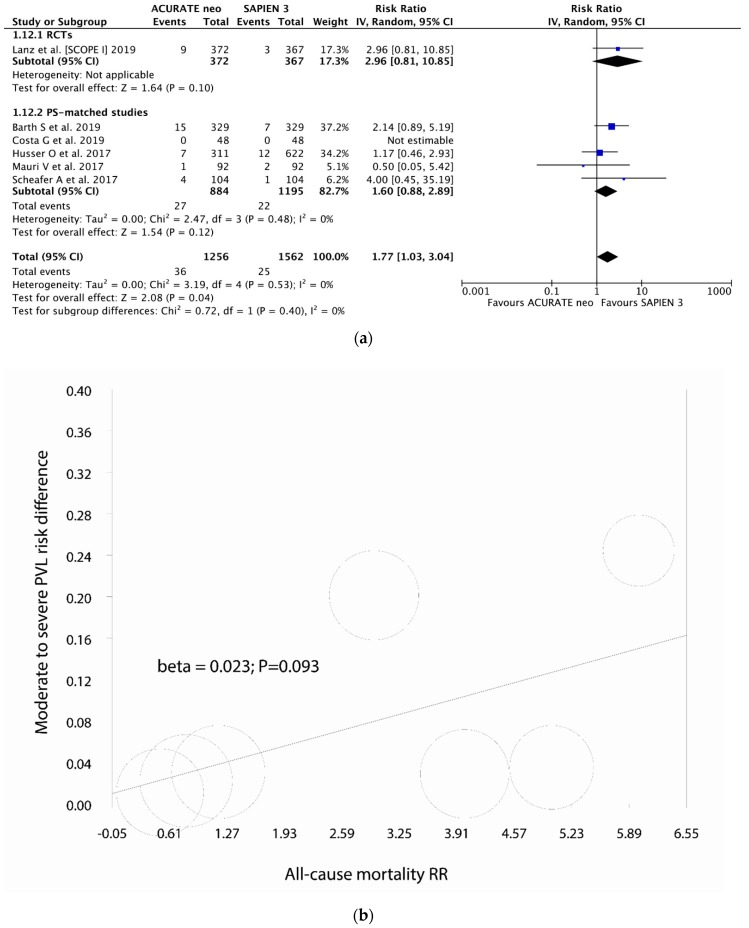

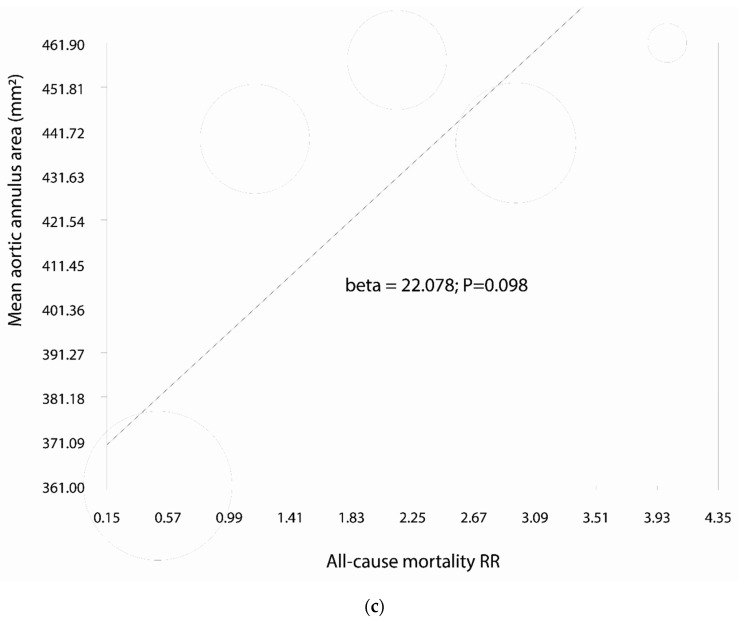
Individual and summary risk ratios with corresponding 95% confidence intervals for the comparison of ACURATE neo vs. SAPIEN 3 in the analysis of (**a**) 30-day all-cause mortality; (**b,c**) meta regression analyses.

**Figure A12:**
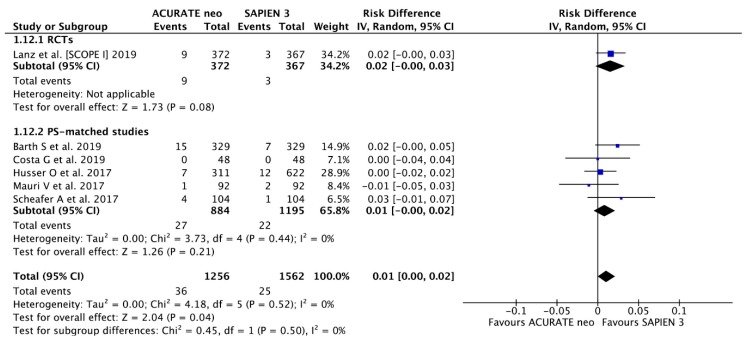
Clinical outcomes. Individual and summary risk ratios with corresponding 95% confidence intervals for the comparison of ACURATE neo and SAPIEN 3 in the analysis of 30-day all-cause mortality taking into account “0 events”.

Six studies reported on 30-day all-cause mortality. Overall, 61 (2.2%) patients died within the first 30 days, with respective rates of 2.9% and 1.6% in ACURATE neo and SAPIEN 3 groups; ACURATE neo was associated with 77% higher 30-day mortality risk (RR 1.77, 95% CI, (1.03, 3.04); *P* = 0.04; *I*^2^ = 0% (Figure 5a and Appendix Figure A12). A random-effects meta-regression was fitted, counter-opposing all-cause mortality risk ratio against the risk difference of moderate-to-severe PVL; there was a trend for higher 30-day mortality rates with higher incidence of moderate-to-severe PVL (beta = 0.023; *P* = 0.093) (Figure 5b); similarly, a meta-regression was fitted with all-cause mortality risk ratio against the mean annulus area in the ACURATE neo arm showing a trend for lower between devices mortality ratio in smaller annuli (beta = 22.078; *P* = 0.098) (Figure 5c).

## 4. Discussion

To the best of our knowledge, this is the first systematic review and meta-analysis of observational trials comparing major procedural, short-term clinical and functional outcomes between the ACURATE neo and SEPIEN 3, the next-generation transcatheter valves designed to minimize shortcomings of the earlier-generation devices. Our analysis, by pooling data from one RCT and five PS-matched studies, demonstrated excellent data regarding short-term performance of both devices. Compared populations of patients were well balanced with respect to baseline characteristics and severity of underlying valvular disease. Main findings of the current study are that the ACURATE neo implantation as compared to SAPIEN 3 was associated with lower transvalvular gradients and lower risk of permanent pacemaker implantation. Other clinical endpoints which included vascular complications, AKI, as well as life threatening and major bleeding; stroke and MIs did not differ between the two groups. The use of ACURATE neo procedures were significantly longer and required a greater amount of contrast volume. Device success and early safety combined endpoints, as defined by VARC-2 criteria, were, however, similar regardless the type of valve implanted. Importantly, the current study revealed significantly higher rates of both mild and moderate-to-severe PVL with ACURATE neo as compared to SAPIEN 3 and the latter were indirectly associated with worse survival observed in ACURATE neo group.

Previous observational studies [15,16,17,18,19,20,21] and, among them, the SAVI-TF (Symetis ACURATE neo Valve Implantation Using Transfemoral Access) registry [29,30] reported on excellent short-term outcomes with low complications and, in particular, PPI rates in ACURATE neo valve attributable to the design of the prosthesis. The particularly low gradients also contributed to the similar or better rates of device success for ACURATE neo and SAPIEN 3 in propensity matched comparisons. Whether the abovementioned benefits would hold true in randomized populations and further translate into improved clinical outcomes was investigated in the Safety and Efficacy of the Symetis ACURATE Neo/TF Compared to the Edwards SAPIEN 3 Bioprosthesis trial (SCOPE I) [14]. Interestingly, the ACURATE neo valve failed to meet noninferiority for its primary endpoint of combined at 30 days against the balloon-expandable SAPIEN 3 (Edwards Lifesciences) valve. Moreover, secondary analyses demonstrated SAPIEN 3 to be superior for the composite safety and efficacy endpoint, driven by less stage 2 or 3 acute kidney injury and less paravalvular leak. Valve dysfunction requiring repeat interventions was also less common at 30 days. In particular, findings on device success need to be addressed, since the rates varied largely between RCT and the remaining PS-matched studies driven by higher patient prosthesis mismatch in the SAPIEN 3 group (*P* < 0.00001). Indeed, median mean transvalvular gradient was lower, and the median mean aortic valve area was larger, in the ACURATE neo, compared to the SAPIEN 3 group, at follow-up echocardiography in the SCOPE I trial. This may have been partially due to the fact that sizing and thus the choice of the valve process were different in the SCOPE I and the remaining studies. Some residual bias despite propensity score matching also cannot be excluded. In fact, Mauri et al. [18] reports on the sizing category was based on perimeter for ACURATE neo and annular area for SAPIEN 3, then all patients received ACURATE neo size S or SAPIEN 3 23 mm. In the study by Husser et al. [17] after PS-matching, there remained a *P* = 0.003 difference in aortic annular area; Schaefer et al. [19] reports aortic annulus size to have presented significant differences for area derived aortic annulus diameter (23.9 ± 2.8 vs. 24.8 ± 2.6 mm; *P* = 0.02) and perimeter-derived aortic annulus diameter (24.5 ± 2.5 vs. 25.3 ± 2.6 mm; *P* = 0.02), which, in consequence, led to oversizing in the ACURATE neo and undersizing SAPIEN 3 (1.5 ± 6.6 vs. −0.9 ± 6.4; *P* = 0.01 for cover index). Further, only in the SCOPE I trial, both the clinical events and functional assessment details were adjudicated by independent core lab. Independently, there were fewer PPI necessary after ACURATE neo in the PS-matched studies; since not confirmed in the SCOPE I, the supra-annular positioning of the valve must have had played, however, a much less important role than expected, and the lower PPI rates originating from skewed valve-size selection and positioning of the valve in the annulus [29]. More importantly, though, SCOPE I trial, by design, excluded over 300 patients with excessive calcification of aortic valve or left ventricular outflow tract (LVOT), which was not the case in remaining studies included in the current analysis. Presence of calcifications in both aortic annulus and LVOT could have accounted for much higher rates of PPI in the SAPIEN 3 arms across included PS-matched studies (average PPI incidence rate of 15.8%) as compared to SCOPE I trial with 9.3% rate, similarly to what has been already demonstrated for SAPIEN 3 in another meta-analysis by the same group [30].

Conversely to the abovementioned, yet still contributing to device success rates, was the higher incidence of moderate-to-severe PVL in the ACURATE neo valve, which was confirmed also in the current meta-analysis. In the next-generation devices, improved by addition an external sealing cuff or a skirt, the frequencies of mild and moderate-to-severe PVL became significantly lower as compared with the earlier-generation valves. The pooled occurrence of more than mild PVL decreased from 6.9% SAPIEN XT to 1.6% in SAPIEN 3 valve, as in a meta-analysis by Ando et al. with 2498 patients [31]. The PARTNER II SAPIEN-3 trial, which assessed early outcomes after TAVR in inoperable, high-risk and intermediate-risk patients with severe aortic stenosis, showed moderate-to-severe PVL in 3.4% and mild in 40.7% of the cases [32]. The abovementioned improvements seen in next-generation devices seem not to be the case with ACURATE neo; in the meta-analysis, we found 11.7% incidence of moderate-to-severe PVL in the ACURATE neo arm, nearly fourfold higher than in SAPIEN 3 and mild PVL in 45.5% cases, translating into 60% increased risk. Unlike the current findings, SAVI TF registry showed 4.1% of >mild PVL in 1000 patients treated with ACURATE neo which is within ranges observable for other devices [33,34,35,36,37,38]. Postdilatation was performed in 44.8% of the patients in that series, and this percentage is also comparable to 40.4%–51.9% in the current analysis, and therefore, theoretically, should not influence the outcome; on the other hand, Barth et al. [15] reports lower >mild PVL rates in one of participating centers (C) that used “zero tolerance of more than mild paravalvular leak” policy and postdilated more frequently than other centers (52.7% as compared to 12.3% and 33.3%), which translated to 3.4% rate of >mild PVL (as compared to 6.0% and 34.1% in the remaining centers). Interestingly, this center was the one to demonstrate highest one-year survival (87.4% (95% CI: 79.6–92.3) compared to 75.4% (95% CI: 60.4–85.3) and 81.3% (95% CI: 70.1–88.6)). Corroborating these estimates on larger scale and also in shorter follow-up, the current meta-analysis found an indirect link between increased rates of >mild PVL and higher mortality in the ACURATE neo arm at 30 days. While the presence of residual >mild PVL has been long shown to be associated with increased mortality in the long-term [39,40], the link between >mild PVL and 30-day mortality appears less clear, particularly for next-generation devices [12]. The abovementioned may be of importance given the fact that acute aortic insufficiency of various degree in patients with prior pure aortic stenosis and diminished LV compliance is often a cause of heart-failure exacerbation early in the sequelae [41].

An indirect link to increased mortality with ACURATE neo, as found also in meta-regression of annular area; indeed, lower between-devices mortality risk ratios between ACURATE neo and SAPIEN 3 were shown in patients with smaller annuli. An important hypothesis generated by present meta-analysis is that ACURATE neo performs differently in this setting; since we could not demonstrate excess of annual ruptures, cardiac tamponades, conversions to surgery or other periprocedural complications in either group, the explanation of this phenomenon remains to be elucidated. 

Several inherent limitations to the current analysis need to be acknowledged; firstly, the majority of included studies are of an observational nature. Despite accounting for differences in the patients’ baseline populations by propensity matching in all of the non-randomized reports, there remain other confounders, like learning curve, operators’ experience and decision as of valve size and type that add to the risk of bias. Indeed, it cannot be refused that ACURATE neo was the preferred valve in smaller aortic annuli in PS-matched studies. Secondly, one study [15] reports on outcomes with both transfemoral ACURATE neo and transapical ACURATE TA systems. While similar in stent design and technological features, there are certain, albeit minor, differences in delivery system and biological tissue used in both devices [42]. Thirdly, only half of included studies reported follow-up longer than one month; paucity of data regarding long-term clinical and functional outcomes significantly impedes interpretation of ACURATE neo and SAPIEN 3 clinical suitability. Lastly, all but one study [14] lacked of an external core lab assessment and adjudication of echocardiographic outcomes. Finally, to better visualize the relative advantages of the contemporary-use valve systems, the results of a second similar study, SCOPE II (NCT03192813), will compare the ACURATE neo to the EVOLUT R system with respect to a composite of all-cause death and stroke at one year.

## 5. Conclusions

Contemporary evidence shows good short-term implantation outcomes of both ACURATE neo and SAPIEN 3 valves, with no differences in combined endpoints of device success and early safety. Implantation of ACURATE neo was associated with lower transvalvular gradients and lower risk of permanent pacemaker implantation. Moderate-to-severe PVL rates were, however, higher in ACURATE neo valve and were indirectly associated with increased 30-day all-cause mortality.

## Figures and Tables

**Table 1:** Valve characteristics and features.

ACURATE neo (Boston Scientific Corporation)	SAPIEN 3 (Edwards Lifesciences)
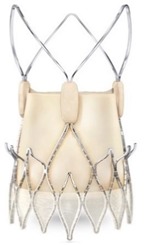	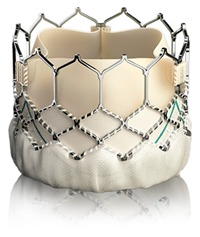
Supra-annular	Intra-annular
Porcine pericardial leaflet tissue	Bovine pericardial leaflet tissue
Self-expanding, deployment in a top-down mechanism of nitinol frame.	Balloon-expandable cobalt-chromium frame
Transfemoral sheath size (valve size)	
18-French for all devices: Small (23 mm), Medium (25 mm), Large (27 mm).	Ready for ultra-low profile: 14 F (20, 23, 26 mm); 16 F (29 mm), 18 F (20, 23, 26 mm), 21 F (29 mm)
Special features	
-Upper and lower crown; -Three stabilization arches; -Outer and inner pericardial skirt.	-Outer sealing and inner skirt at the inflow

**Table 2:** Baseline characteristics of included studies.

Study	Barth S et al. 2019 [15]		Costa et al. 2019 [16]		Husser O et al. 2017 [17]		Lanz J et al. 2019 [14]		Mauri V et al. 2017 [18]		Schaefer A et al. 2017 [19]	
	ACURATE neo	SAPIEN 3	ACURATE neo	SAPIEN 3	ACURATE neo	SAPIEN 3	ACURATE neo	SAPIEN 3	ACURATE neo	SAPIEN 3	ACURATE neo	SAPIEN 3
Study period	2012–2016		09.2014–02.2018		01.2014–01.2016		02.2017–02.2019		02.2014–08.2016		2012–2016	
Design	MC, RCS, PM		SC, RCS, PM		MC, RCS, PM		MC, RCT		MC, RCS, PM		SC, RCS, PM	
Number of pts.	329	329	48	48	311	622	372	367	92	92	104	104
Age	81.0 ± 5.0	81.0 ± 6.0	82.3 ± 3.8	83.3 ± 2.3	81.0 ± 6.0	81.0 ± 6.0	82.6 ± 4.3	83.0 ± 3.9	82.8 ± 6.5	81.9 ± 5.3	81.7 ± 5.5	81.2 ± 6.2
Female (%)	NR		70.8	68.8	60.8	55.3	59.0	55.0	92.4	92.4	69.2	65.4
BMI (kg/m^2^)	28.7 ± 5.5	28.4 ± 5.8	27.8 ± 4.6	27.1 ± 3.9	27.0 ± 5.0	27.0 ± 5.0	27.3 ± 4.4	27.9 ± 4.7	27.3 ± 5.5	26.0 ± 4.7	27.1 ± 5.1	26.8 ± 5.0
STS-PROM (%)	NR		4.0 ± 3.3	3.8 ± 1.7	NR		3.7 ± 1.8	3.7 ± 1.9	NR		5.8 ± 3.8	5.4 ± 3.6
Logistic EuroSCORE (%)	18.8 ± 14.7	19.1 ± 13.6	NR	NR	18.0 ± 10.0	18.0 ± 12.0	NR	NR	16.2 ± 8.8	16.6 ± 8.8	15.9 ± 9.3	13.7 ± 9.0
NYHA III/IV (%)	79.0	78.1	NR		256	489	77.0	73.0	NR		86.5	88.5
EF (%)	53.0 ± 13.0	54.0 ± 15.0	54.5 ± 9.7	56.1 ± 9.7	NR	NR	56.4 ± 11.1	57.1 ± 10.7	59.0 ± 8.0	59.0 ± 10.0	NR	NR
EF < 35% (%)	9.4	10.3	NR		5.8	5.5	NR		NR		26.0 *	22.1 ^1^
Mean aortic gradient (mmHg)	44.0 ± 15.0	45.0 ± 14.0	51.3 ± 14.5	51.3 ± 17.2	45.0±15.0	44.0 ± 16.0	42.9 ± 17.2	41.5 ± 15.1	46.0 ± 16.0	47.0 ± 16.0	35.9 ± 16.6	37.6 ± 16.7
Aortic annulus diameter (mm)	21.0 ± 2.0	21.0 ± 3.0	NR		NR		23.6 ± 1.6	23.7 ± 1.6	NR		24.5 ± 2.5	25.3 ± 2.6
Access site (%)	TF 74.5, TA 25.5	TF 75.7, TA 24.3	TF 100.0	TF 100.0	TF 100.0	TF 100.0	TF 99.0, TA <1.0	TF 100.0	TF 100.0	TF 100.0	TF 100.0	TF 100.0
VARC-2 outcomes definitions	yes		Yes		yes		yes		yes		yes	
Follow-up (months)	10.8 ± 9.7	12.2 ± 9.9	12		1		1		12.7 ± 2.6		1	

^1^ <44% EF; RCT, randomized control trial; SC, single center; MC, multi center; RCS, retrospective cases series; PM, propensity matching; NYHA, New York Heart Association; STS-PROM, Society of Thoracic Surgeons Predicted Risk of Mortality; EuroSCORE, European System for Cardiac Operative Risk Evaluation; VARC, Valve Academic Research Consortium; EF, ejection fraction; TF, trans femoral; TA, trans apical; NR, not reported. In bold are highlighted the variables that differed significantly between study groups.

## Appendix A

**Table A1:** Checklist for meta-analyses of observational studies.

Item No.	Recommendation	Reported on Page No.
Reporting of background should include		
1	Problem definition	2
2	Hypothesis statement	NA
3	Description of study outcome(s)	3–11
4	Type of exposure or intervention used	5
5	Type of study designs used	5
6	Study population	5
Reporting of search strategy should include		
7	Qualifications of searchers (e.g., librarians and investigators)	Title page
8	Search strategy, including time period included in the synthesis and key words	4, Figure 1
9	Effort to include all available studies, including contact with authors	5
10	Databases and registries searched	5
11	Search software used, name and version, including special features used (e.g., explosion)	NA
12	Use of hand searching (e.g., reference lists of obtained articles)	5
13	List of citations located and those excluded, including justification	NA
14	Method of addressing articles published in languages other than English	NA
15	Method of handling abstracts and unpublished studies	NA
16	Description of any contact with authors	NA
Reporting of methods should include		
17	Description of relevance or appropriateness of studies assembled for assessing the hypothesis to be tested	NA
18	Rationale for the selection and coding of data (e.g., sound clinical principles or convenience)	NA
19	Documentation of how data were classified and coded (e.g., multiple raters, blinding and interrater reliability)	NA
20	Assessment of confounding (e.g., comparability of cases and controls in studies where appropriate)	Table A2
21	Assessment of study quality, including blinding of quality assessors, stratification or regression on possible predictors of study results	Table A2
22	Assessment of heterogeneity	3
23	Description of statistical methods (e.g., complete description of fixed or random effects models, justification of whether the chosen models account for predictors of study results, dose-response models, or cumulative meta-analysis) in sufficient detail to be replicated	3
24	Provision of appropriate tables and graphics	yes
Reporting of results should include		
25	Graphic summarizing individual study estimates and overall estimate	Figure 3, Figure 4 and Figure 5
26	Table giving descriptive information for each study included	Table 2
27	Results of sensitivity testing (e.g., subgroup analysis)	NA
28	Indication of statistical uncertainty of findings	13–14
Reporting of discussion should include		
29	Quantitative assessment of bias (e.g., publication bias)	NA
30	Justification for exclusion (e.g., exclusion of non-English language citations)	Figure 1
31	Assessment of quality of included studies	13, Table A2
Reporting of conclusions should include		
32	Consideration of alternative explanations for observed results	11–13
33	Generalization of the conclusions (i.e., appropriate for the data presented and within the domain of the literature review)	14
34	Guidelines for future research	NA
35	Disclosure of funding source	Title page

From: Stroup DF, Berlin JA, Morton SC, et al. for the Meta-Analysis of Observational Studies in Epidemiology (MOOSE) Group. Meta-Analysis of Observational Studies in Epidemiology. A Proposal for Reporting. JAMA 2000; 283:2008-2012.

**Table A2:** Publication bias analysis.

Study (RCT)	Random sequence generation (selection bias)	Allocation concealment (selection bias)	Blinding of participants and personnel (performance bias)	Blinding of outcome assessment (detection bias)		Incomplete outcome data (attrition bias)	Selective reporting (reporting bias)	Other bias
**Lanz et al. [SCOPE I] 2019 [14]**	Low	Unclear	High	Low		Low	Low	Low
**Study (PS-matched studies)**	**Bias due to confounding**	**Bias in selection of participants into the study**	**Bias in measurement of interventions**	**Bias due to departures from intended interventions**	**Bias due to missing data**	**Bias in measurement of outcomes^1^**	**Bias in selection of reported result**	**Overall bias**
Barth S et al. 2019 [15]	Serious	Serious	Low	Low	Low	Serious	Low	Moderate
Costa G et al. 2019 [16]	Serious	Low	Low	Low	Low	Serious	Low	Moderate
Husser O et al. 2017 [17]	Serious	Low	Low	Low	Low	Serious	Low	Moderate
Mauri V et al. 2017 [18]	Serious	Low	Low	Low	Low	Serious	Low	Moderate
Scheafer A et al. 2017 [19]	Serious	Low	Low	Low	Low	Serious	Low	Moderate

^1^ When multiple outcomes were reported for a study, the highest level of bias at the outcome level is reported in the table.

**Table A3:** Inclusion and exclusion criteria. Choice of procedure and valve-type.

Study [ref]	Inclusion criteria	Exclusion criteria	Selection criteria for the procedure	Selection criteria for the valve
Barth S et al. 2019 [15]	Patients received either the ACURATE/ACURATE neo prostheses (*n* = 591) or the SAPIEN 3 prosthesis (*n* = 715).	Through nearest neighborhood matching with exact allocation for access route and center, pairs of 329 patients (250 transfemoral, 79 transapical) per group were determined.	Not reported.	Not reported.
Costa et al. 2019 [16]	All the patients treated with SAPIEN 3, Evolut R, or ACURATE neo, which could have indifferently received all the three devices according to manufacturer sizing indications.	Patients who did not performed pre-TAVI multi-detector computed tomography assessment (n = 169), patients who had a valve-in- valve implantation in a failed aortic bioprosthesis (n = 21), patients with bicuspid aortic valve (n = 28), and pure aortic regurgitation (n = 1).	Not reported.	Not reported.
Husser O et al. 2017 [17]	Patients with symptomatic, severe stenosis of the native aortic valve were treated with transfemoral TAVI using ACURATE neo (*n* = 311) or SAPIEN 3 (*n* = 810) at 3 centers in Germany.	Not reported.	The interdisciplinary heart team discussed all cases and consensus was achieved regarding the therapeutic strategy.	The interdisciplinary heart team discussed all cases and consensus was achieved regarding the therapeutic strategy.
Lanz J et al. 2019 [14]	Patients aged 75 years or older. With severe aortic stenosis defined by an aortic valve area (AVA) < 1 cm^2^ or AVA indexed to body surface area of < 0·6 cm^2^/m^2^. Symptomatic (NYHA functional class > I, angina or syncope). At increased risk for mortality if undergoing SAVR as determined by: - the heart team OR - an STS-PROM score > 10% OR - a Logistic EuroSCORE > 20%. Heart team agrees on eligibility for participation. Aortic annulus perimeter 66–85 mm AND area 338–573 mm^2^ based on multi-slice computed tomography. Minimum diameter of arterial aorto-iliac-femoral axis on one side: ≥5·5 mm. Patient understand the purpose, potential risks and benefits of the trial, is able to provide written informed content and willing to participate in all parts of the follow-up.	-Non-valvular, congenital or non-calcific acquired aortic stenosis, uni- or bicuspid aortic valve. -Anatomy not appropriate for transfemoral TAVR due to degree or eccentricity of calcification or tortuosity of aorto- and iliac-femoral arteries. -Pre-existing prosthetic heart valve in aortic or mitral position. -Emergency procedures, cardiogenic shock (vasopressor dependence, mechanical hemodynamic support), or severely reduced left ventricular ejection fraction (< 20%). -Concomitant planned procedure except for percutaneous coronary intervention. -Stroke or myocardial infarction (except type 2) in prior 30 days. -Planned non-cardiac surgery within 30 days after TAVR. -Severe coagulation conditions, inability to tolerate anticoagulation/antiplatelet therapy. -Evidence of intra-cardiac mass, thrombus or vegetation. -Active bacterial endocarditis or other active infection. -Hypertrophic cardiomyopathy with or without obstruction. -Contraindication to contrast media or allergy to nitinol. -Participation in another trial leading to deviations in the preparation and conduction of the intervention or the post-implantation management.	The heart team or an STS-PROM score > 10% or a Logistic EuroSCORE > 20%. Heart team agrees on eligibility for participation.	Patients were randomly assigned in a 1:1 ratio to undergo TAVI with either the ACURATE neo or the SAPIEN 3 system.
Mauri V et al. 2017 [18]	Inclusion criteria were small annular dimension defined as an annulus area <400 mm^2^ and transfemoral TAVI with either an ACURATE neo size S or an Edwards SAPIEN 3 size 23 mm.	Not reported.	Eligibility of the individual candidate for TAVI had been decided within the local institutional heart team.	Prosthesis selection was at the discretion of the operating physicians at each center.
Schaefer A et al. 2017 [19]	A consecutive series of 104 patients received transfemoral TAVI using the ACURATE neo for treatment of severe symptomatic calcified aortic stenosis (study group) between 2012 and 2016. For comparative assessment, a matched control group of 104 patients treated by transfemoral TAVI using the Edwards SAPIEN 3 during the same time frame (2014 to 2016) was retrieved from dedicated hospital database containing a total of 1326 TAVI patients (210 SAPIEN 3 patients).	Patients unsuitable for a retrograde transfemoral approach and all valve-in-valve procedures were excluded from analysis.	Allocation of patients to TAVI followed current international recommendations after consensus of the local dedicated heart team.	Not reported.

**Table A4:** Patients’ baseline characteristics.

Study [ref]	Intervention	HT (%)	DM (%)	PVD (%)	CKI (%)	COPD (%)	PM/ICD (%)	AF (%)	CAD (%)	MI history (%)	Stroke history (%)	Heart surgery history (%)	NYHA III/IV (%)	LVEF (%)	Mean aortic gradient (mmHg)	Aortic valve area (cm^2^)	Aortic annulus diameter (mm)
Barth S et al. 2019 [15]	ACURATE neo	93.3	36.8	**NR**	**2.7**	**15.8**	**NR**	**38.0**	**NR**	**NR**	**14.0**	**14.9**	**79.0**	**53.0 ± 13.0**	**44.0 ± 15.0**	**0.68 ± 0.18**	21.0 ± 2.0
	SAPIEN 3	93.0	35.0	NR	2.7	14.9	NR	38.7	NR	NR	14.6	14.6	78.1	54.0 ± 15.0	45.0 ± 14.0	0.67 ± 0.17	21.0 ± 3.0
Costa et al. 2019 [16]	ACURATE neo	89.6	18.8	6.3	4.2	20.8	NR	12.5	NR	14.6	2.1	6.3	NR	54.5 ± 9.7	51.3 ± 14.5	NR	NR
	SAPIEN 3	89.6	27.1	4.2	2.1	14.6	NR	12.5	NR	14.6	4.2	2.1	NR	56.1 ± 9.7	51.3 ± 17.2	NR	NR
Husser O et al. 2017 [17]	ACURATE neo	NR	33.1	10.6	2.3	13.5	9.0	24.8	61.1	10.0	13.8	10.6	82.3	NR	45.0 ± 15.0	NR	NR
	SAPIEN 3	NR	32.3	11.3	1.9	17.8	10.0	26.2	62.7	10.1	12.5	8.7	78.6	NR	44.0 ± 16.0	NR	NR
Lanz J et al. 2019 [14]	ACURATE neo	92.0	29.0	12.0	4.0	9.0	12.0	36.0	59.0	10.0	13.0	9.0	77.0	56.4 ± 11.1	42.9 ± 17.2	0.7 ± 0.2	23.6 ± 1.6
	ACURATE neo	91.0	32.0	11.0	5.0	12.0	10.0	37.0	60.0	13.0	13.0	9.0	73.0	57.1 ± 10.7	41.5 ± 15.1	0.7 ± 0.2	23.7 ± 1.6
Mauri V et al. 2017 [18]	SAPIEN 3	NR	NR	NR	NR	NR	NR	NR	NR	NR	NR	NR	NR	59.0 ± 8.0	46.0 ± 16.0	NR	NR
	ACURATE neo	NR	NR	NR	NR	NR	NR	NR	NR	NR	NR	NR	NR	59.0 ± 10.0	47.0 ± 16.0	NR	NR
Schaefer A et al. 2017 [19]	SAPIEN 3	85.6	27.9	16.3	NR	17.3	NR	34.6	59.6	NR	14.4	9.6	86.5	NR	35.9 ± 16.6	0.8 ± 0.2	24.5 ± 2.5
	ACURATE neo	93.3	26.0	13.5	NR	20.2	NR	32.7	57.7	NR	11.5	5.8	88.5	NR	37.6 ± 16.7	0.8 ± 0.2	25.3 ± 2.6

HT, hypertension; DM, diabetes mellitus; PVD, peripheral vascular disease; CKI, chronic kidney injury; COPD, chronic obstructive pulmonary disease; PM/ICD, pacemaker/implantable cardioverter-defibrillator; AF, atrial fibrillation; CAD, coronary artery disease; MI, myocardial infarction; LVEF, left ventricle ejection fraction; NR, not reported. In bold are highlighted the variables that differed significantly.

**Table A5:** Procedural characteristics.

Study [ref]	Intervention	Anesthesia (%)	Access Site (%)	Valve sizes Implanted (%), (Mean ± SD)	Pre-Dilatation (%)	Post-Dilatation (%)	Contrast Volume (mL)	Fluoroscopy Time (min)	Procedure Duration (min)
Barth S et al. 2019 [15]	ACURATE neo	general 96.0, conscious sedation 4.0	femoral 74.5, apical 25.5	S NR M NR L NR (25.0 ± 2.0)	97.6	40.4	128 ± 54	9.2 ± 4.4	62.0 ± 24.0
	SAPIEN 3	general 96.4, conscious sedation 3.6	femoral 75.7, apical 24.3	23 mm NR 26 mm NR 29 mm NR (25.0 ± 2.0)	52.1	11.6	106 ± 43	8.5 ± 4.9	59.0 ± 26.0
Costa et al. 2019 [16]	ACURATE neo	NR	femoral 100	NR	NR	NR	NR	NR	NR
	SAPIEN 3		femoral 100	NR	NR	NR	NR	NR	NR
Husser O et al. 2017 [17]	ACURATE neo	general 52.7, conscious sedation 47.3	femoral 100	S 30.9 M 40.2 L 28.9	95.8	42.1	115.0 ± 54.0	10.0 ± 6.0	55.0 ± 30.0
	SAPIEN 3	general 54.0, conscious sedation 46.0	femoral 100	23 mm 43.9 26 mm 41.6 29 mm 14.5	74.3	23.8	104.0 ± 53.0	11.0 ± 5.9	54.0 ± 24.0
Lanz J et al. 2019 [14]	ACURATE neo	general 25.0, conscious sedation 75.0	femoral 99.0, other 1.0	S 20.0 M 43.0 L 34.0	88.0	52.0	136.0 ± 55.6	NR	53.2 ± 26.5
	SAPIEN 3	general 23.0, conscious sedation 77.0	femoral 99.0, other 1.0	23 mm 39.0 26 mm 55.0 29 mm 5.0	23.0	48.0	110 ± 45.9	NR	46.0 ± 25.9
Mauri V et al. 2017 [18]	ACURATE neo	general 100.0	femoral 100	S 100.0	94.6	31.5	NR	NR	NR
	SAPIEN 3		femoral 100	23 mm 100.0	31.5	6.5	NR	NR	NR
Schaefer A et al. 2017 [19]	ACURATE neo	conscious sedation 47.1% general 52.9%	femoral 100	S 35.6 M 38.5 L 25.9	90.3	47.6	162.6 ± 70.3	19.3 ± 9.4	94.0 ± 46.9
	SAPIEN 3	conscious sedation 34.6% general 65.4%	femoral 100	23mm 40.4 26mm 49.0 29mm 10.6	53.8	20.2	154.8±73.0	19.4±9.1	94.8±38.0

NR, not reported.

**Table A6:** VARC-2 derived permanent pacemaker implantation criteria quality appraisal.

Study [ref]	Presence of Pacemaker at Baseline Reported	Precision of the Indication Reported	Days Post TAVR for PPI Reported
Barth S et al. 2019 [15]	No	no	In-hospital
Costa et al. 2019 [16]	Yes	no	NA
Husser O et al. 2017 [17]	Yes	no	In-hospital and 30 days
Lanz J et al. 2019 [14]	Yes	no	30 days
Mauri V et al 2017 [18]	No	no	30 days
Schaefer A et al. 2017 [19]	No	Atrioventricular block Grade 3 or rapid progressive left bundle branch block	In-hospital

TAVR, transcatheter aortic valve replacement; PPI, permanent pacemaker implantation; NA, not available.
